# Robotic-assisted versus conventional/single-incision laparoscopic cholecystectomy for benign gallbladder disease: A systematic review and meta-analysis

**DOI:** 10.1097/MD.0000000000042493

**Published:** 2025-05-23

**Authors:** Kezhong Tang, Wei Zhou, Yizhao Zhou, Yongzhou Li, Li Liao, Xin Dong

**Affiliations:** a Department of Surgery, Second Affiliated Hospital, Zhejiang University School of Medicine, Hangzhou, PR China; b Department of Surgery, First People’s Hospital of Pinghu District, Jiaxing, PR China; c Department of Pharmacy, Hangzhou Children’s Hospital, Hangzhou, PR China.

**Keywords:** benign gallbladder disease, laparoscopic cholecystectomy, robotic-assisted, single-incision

## Abstract

**Background:**

Robotic surgery has shown great advantages in many complicated endoscopic operations compared with laparoscopic surgery. However robotic-assisted cholecystectomy (RAC) is still a controversial issue compared with conventional/single-incision laparoscopic cholecystectomy (CLC/SILC). The aim of this study was to compare the safety and efficacy of RAC with CLC/SILC for benign gallbladder disease.

**Methods:**

Embase, Medline, Pubmed, and Cochrane library databases were searched to obtain comparative studies evaluating the safety and efficacy between RAC and CLC/SILC. Only randomized trials and non-randomized studies with propensity score matching were included. Statistical analysis was performed through Stata software, random effects models were applied.

**Results:**

A total of 17 studies, including 12 studies for RAC versus CLC and 5 studies for RAC versus SILC, were included in the study. A total of 75,866 patients were included in the study, of whom 37,471 patients underwent RAC, 38,123 patients underwent CLC, and 272 patients underwent SILC. Compared with CLC/SILC, RAC significantly increased operative time (standardized mean difference [SMD] 0.79 minutes; 95% confidence interval [CI] 0.30, 1.28) and hospitalization costs (SMD 1.82; 95% CI 0.27, 3.36). At the same time, RAC significantly reduced conversion rate (relative risk [RR] 0.58; 95% CI 0.52, 0.63). There were no significant differences between the groups in the length of hospital stay (SMD 0.04; 95% CI ‐0.02, 0.11), intraoperative complications (RR 0.99; 95% CI 0.61, 1.59), estimate blood loss (SMD 0.03; 95% CI ‐0.19, 0.25), and incisional hernia (RR 2.51; 95% CI 0.69, 9.10).

**Conclusion:**

RAC is a safe and effective procedure with lower conversion rate and comparable rate of complications compared with CLC/SILC.

## 1. Introduction

Cholecystectomy is a well-established and commonly performed procedure for benign gallbladder diseases.^[1]^ Laparoscopic cholecystectomy, including conventional laparoscopic cholecystectomy (CLC) and single-incision laparoscopic cholecystectomy (SILC), was considered a gold standard treatment for benign gallbladder diseases.^[2,3]^ Recently, robotic-assisted surgery has obtained increased popularity and applicability in general surgery and may provide better outcomes than laparoscopic surgery in certain gastrointestinal surgery.^[4]^ Robotic-assisted cholecystectomy (RAC) was considered a safe and efficacious treatment. Compared with CLC/SILC, RAC has several technical advantages including more precise application of energy, 3D high-definition vision, enhanced instrument articulation and improved cosmetics with single port approach.^[5,6]^ However, several studies have mentioned the drawbacks of RAC including longer operative time and higher hospitalization costs, the use of RAC is still controversial and need further evaluation.

Several meta-analyses comparing RAC and LC had been published and provided some significant insights, such as longer operation time, higher hospitalization costs and higher incidence of incisional hernia.^[7,8]^ However, as new high-quality studies have been published recently, some insights about RAC and LC published before need further reevaluation. For example, a recent study reported RAC had a decreased prevalence of conversion rate compared with CLC/SILC.^[9]^ Hence, this meta-analysis selected high-quality studies including randomized trials (RCT) and non-randomized studies with propensity score matching (PSM) to systematically assess the safety and efficacy of RAC compared with CLC/SILC for benign gallbladder disease.

## 2. Materials and methods

### 2.1. Literature search strategy

An electronic literature search was undertaken using Embase, Medline, Pubmed, and Cochrane library databases up to February 2025. Additional articles were identified by hand searching. Search terms included “single-incision,” “single-port,” “single access,” “robotic,” “da Vinci,” and “laparoscopic cholecystectomy.” Two authors Tang and Zhou performed the electronic search independently in February 2025. Abstracts of the literatures were reviewed by authors Tang and Zhou to determine their suitability for inclusion in the pooled analysis. Any discordances regarding study inclusion between these 2 authors were settled in discussion with a third independent author. Assessment according to the Cochrane Collaboration’s tool for evaluating risk of bias in randomized and non-randomized trials were performed and showed in Supplementary Materials, Supplemental Digital Content, https://links.lww.com/MD/O965. The PROSPERO registration number of this study was CRD420250650204.

### 2.2. Study inclusion and exclusion criteria

Publications were included in this review if they meet the following criteria:

(1) RCTs and observational studies with PSM.
(2) Comparative study about RAC and CLC/SILC.
(3) The study should report at least one of the outcomes of interest.

Publications were excluded if they met any of the following criteria:

(1) Not for human study.
(2) No original data are available for extraction.
(3) Case reports, conference abstracts, meta-analysis and reviews.

### 2.3. Data extraction

A standardized data collection form was used to extracted the following information: first author’s name, publication year, country, study design, type of LC received, sex, age range, body mass index, number of cases, the total sample size, and surgical indications. When a study presented models that adjusted for different numbers of control variables, data were extracted from the model that adjusted for the largest number of variables. In the situation in which authors from the same institution had published a primary paper and then an updated analysis with a larger patient cohort was included in the analysis.

### 2.4. Statistical analysis

Two independent reviewers extracted data from the selected articles by using a predefined data extraction form, including operative time, hospitalization time, intraoperative complications, estimated blood loss, occurrence of port-site hernia, bile duct injury, conversion rate and hospitalization costs. To estimate relative risk (RR) and its variance, this was extracted from the study directly or required additional calculation depending on the method of data being presented: the number of patients with outcomes of interest in RAC group and CLC/SILC group. The same method was used to estimate standardized mean difference (SMD) in analysis of continuous variables.

Meta-analysis of data was conducted using a random effects model. Publication bias was explored graphically with funnel plots to detect asymmetry and any outliers. Inter-study heterogeneity was assessed using the *x*^2^ statistic and the *I*^2^ value to measure the degree of variation not attributable to chance alone. This was graded as low (*I*^2^ < 25%), moderate (*I*^2^ = 25%–75%), or high (*I*^2^ > 75%). The significance level was set at *P* < .05. This meta-analysis is exempt from ethical approval as the analysis involves only already published and anonymized data.

## 3. Results

### 3.1. Search results

**Figure 1.**
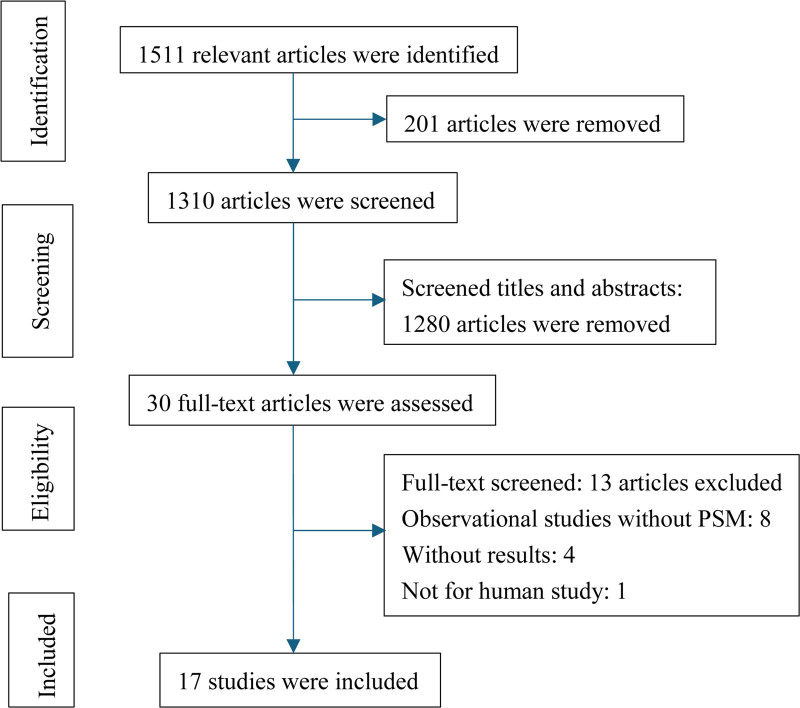
The PRISMA flowchart of literature research.

Figure 1 shows the literature search flowchart. During the literature search we found 1511 studies. Two hundred one articles were removed because of duplication. After reviewing the titles and abstracts we found 1280 articles to be not eligible as they were review articles, editorials, meta-analysis or not focusing on the review topic, and others not meeting the inclusion criteria. We identified 30 articles as potentially eligible for this review. However, 4 of these articles were lack of outcomes of interest. 1 was not for human study and 8 of them were observational studies without PSM. We finally selected 17 eligible articles, including 4 RCTs^[10–13]^ and 13 non-RCT^[9,14–25]^ with PSM (Fig. 1).

### 3.2. Characteristics of the studies

In this meta-analysis, we included 17 studies to evaluate the safety and effectiveness of RAC compared with CLC/SILC. In Table 1, we report the main characteristics of these studies. A total of 75,866 patients involved in the study, including 4 RCTs and 13 non-RCTs, of which 37,471 patients submitted to RAC, 38,123 patients to CLC and 272 patients to SILC. Of 17 studies included, 7 studies were from USA,^[9,12,17,18,20,23,24]^ 4 from South Korea,^[16,21,22,25]^ 3 from Switzerland,^[13–15]^ 1 from China,^[10]^ Italy,^[11]^ and Lithuania,^[19]^ respectively. Detailed information about female rate, mean age and body mass index were shown in Table 1.

**Table 1:** Baseline characteristics of studies included in the meta-analysis.

Study	Year	Country	Study design	Type of RAC	Type of LC	No. of patients RAC/LC	% of female RAC/LC	Mean age, years RAC/LC	Mean BMI (kg/m^2^) RAC/LC	Surgical indications
Zhou et al^[10]^	2006	China	RCT	MIRC	CLC	20/20	40/35	32–47/30–50	NA	Benign disease
Breitenstein et al^[14]^	2008	Switzerland	n-RCT	RAC	CLC	50/50	76/74	53.2 ± 17.3/51.7 ± 15.9	28.2 ± 6.0/27.7 ± 8.4	Cholecystolithiasis
Pietrabissa et al^[11]^	2015	Italy	RCT	SIRC	CLC	30/30	NA	NA	NA	Gallbladder lithiasis or polyps
Kudsi et al^[12]^	2016	USA	RCT	SIRC	CLC	83/53	78/92	46.8 ± 15.5/46.5 ± 17.3	30.4 ± 6.5/31.7 ± 6.7	Chronic cholecystitis, cholelithiasis/symptomatic cholelithiasis
Hagen et al^[15]^	2017	Switzerland	n-RCT	SIRC	CLC	99/99	73/73	47.4 ± 12.6/47 ± 14	26.2 ± 4.2/26.3 ± 4.9	Benign, noninflammatory disease
Grochola et al^[13]^	2018	Switzerland	RCT	SIRC	SILC	30/30	67/53	52.4/51.5	27.3 ± 3.9/27.3 ± 4.2	Cholecystolithiasis; GB polyp
Ghanem et al^[18]^	2020	USA	n-RCT	SIRC	SILC	71/71	68/63	50 ± 17/52 ± 20	31.5 ± 7.3/31.0 ± 6.8	NA
Han et al^[16]^	2020	South Korea	n-RCT	SIRC	SILC	104/104	72/75	44.88 ± 9.56/46.86 ± 11.47	23.61 ± 3.73/23.7 ± 3.07	Cholelithiasis-related problems
Kane et al^[17]^	2020	USA	n-RCT	RAC	CLC	106/1060	72/70	41.5/43	30.1/30.9	NA
Samalavicius et al^[19]^	2021	Lithuania	n-RCT	RAC	CLC	20/20	60/70	49.5 ± 18.6/55.1 ± 13.3	26.9 ± 3.3/26.5 ± 3.1	Cholelithiasis-related problem
Lee et al^[22]^	2022	South Korea	n-RCT	SIRC	SILC/CLC	39/32/86	74.4/75/57	44.9 ± 11.4/45.9 ± 14/50.9 ± 15.1	24.2 ± 3.2/24.7 ± 3.6/26 ± 4.3	Benign disease
Campbell et al^[20]^	2023	USA	n-RCT	RAC	CLC	9996/9996	NA	NA	NA	Cholelithiasis-related problems (emergent or urgent cholecystectomy)
Jang et al^[21]^	2023	South Korea	n-RCT	SIRC	CLC	117/117	58/62	48.2 ± 9.9/47.9 ± 12	24.5 ± 3.3/24.4 ± 3.9	Cholelithiasis-related problems, adenomyomatosis, GB polyp
Lunardi et al^[9]^	2024	USA	n-RCT	RAC	CLC	26,241/26,241	64.4/64.8	NA	NA	Acute cholecystitis, cholangitis, gallbladder disorders
Klein et al^[23]^	2024	USA	n-RCT	RAC	CLC	130/130	67.7/70.8	48.66 ± 17.08/45.33 ± 19.48	NA	Acute cholecystitis
Svetanoff et al^[24]^	2024	USA	n-RCT	SIRC	CLC	25/25	68/60	15.7 (14.7, 17.3)/15.3 (14.5, 16.9)	NA	Acute cholecystitis and/or choledocholithiasis
Park et al^[25]^	2025	South Korea	n-RCT	SIRC	SILC	30/50/126	60/62/61	46.9 ± 11.7/47.7 ± 8.6/50.2 ± 14.8	24.2 ± 3.2/24.5 ± 3.3/24.1 ± 3.6	Cholelithiasis-related problems, adenomyomatosis, GB polyp

BMI = body mass index, CLC = conventional laparoscopic cholecystectomy, GB = gallbladde, MIRC = multi-incision robotic-assisted cholecystectomy, NA = not available, n-RCT = non-randomized-controlled trial, RAC = robotic-assisted cholecystectomy, RCT = randomized-controlled trial, SILC = single-incision laparoscopic cholecystectomy, SIRC = single-incision robotic-assisted cholecystectomy.

Most of the studies included patients with diagnosis of chronic cholelithiasis and gallbladder polyps, which is considered easier to perform cholecystectomy. Four studies^[9,20,23,24]^ included patients with acute cholecystitis. Two studies^[17,18]^ did not describe the patients’ diagnosis.

### 3.3. Operative times

**Figure 2.**
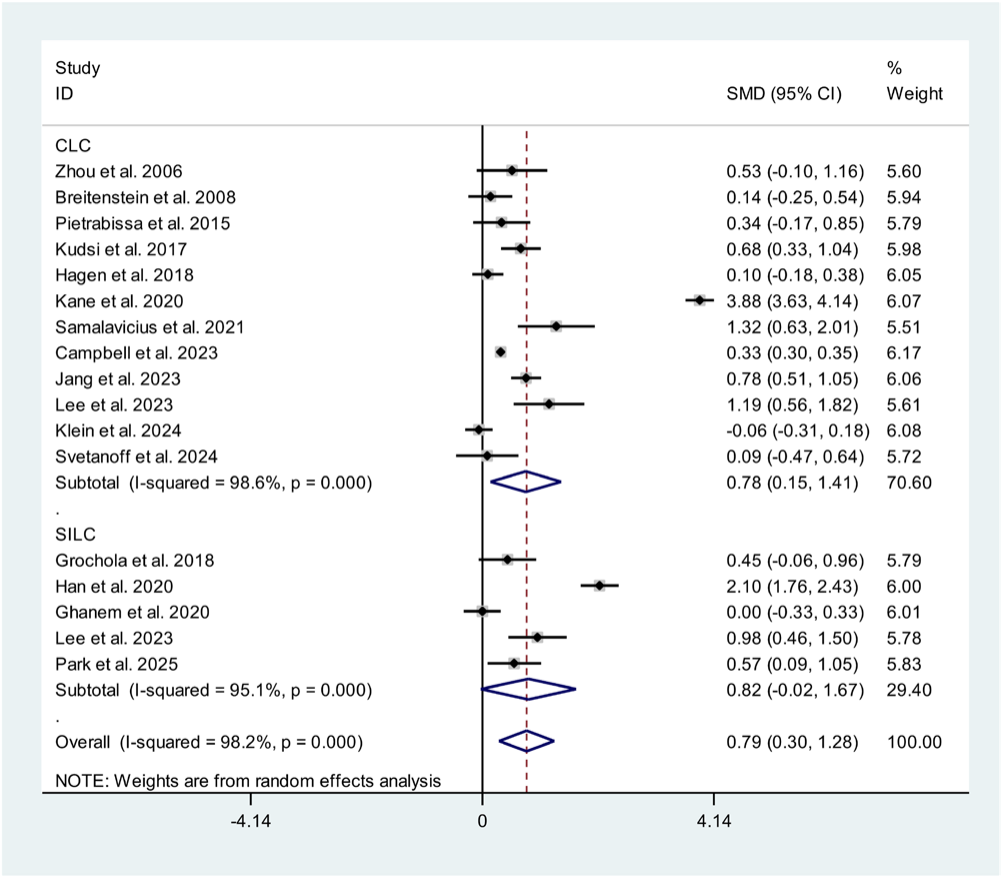
Meta-analysis forest plot concerning operative time.

Sixteen studies, including 4 RCTs^[10–13]^ and 12 non-RCTs,^[14–25]^ reported the operative time. A subgroup analysis for RAC compared with CLC and SILC respectively was performed. Compared with CLC, RAC need a significantly longer operative time (SMD 0.78 minutes; 95% confidence interval [CI] 0.15, 1.41) with a high heterogeneity (*P* < .01, *I*^2^ = 98.6%). In SILC group, a similar result was obtained (SMD 0.82 minutes; 95% CI ‐0.02, 1.67; *P* < .01, *I*^2^ = 95.1%) though without significant differences. The pooled data showed RAC need a significantly longer operative time (SMD 0.79 minutes; 95% CI 0.30, 1.28) compared with CLC/SILC (Fig. 2).

### 3.4. Length of hospital stay

**Figure 3.**
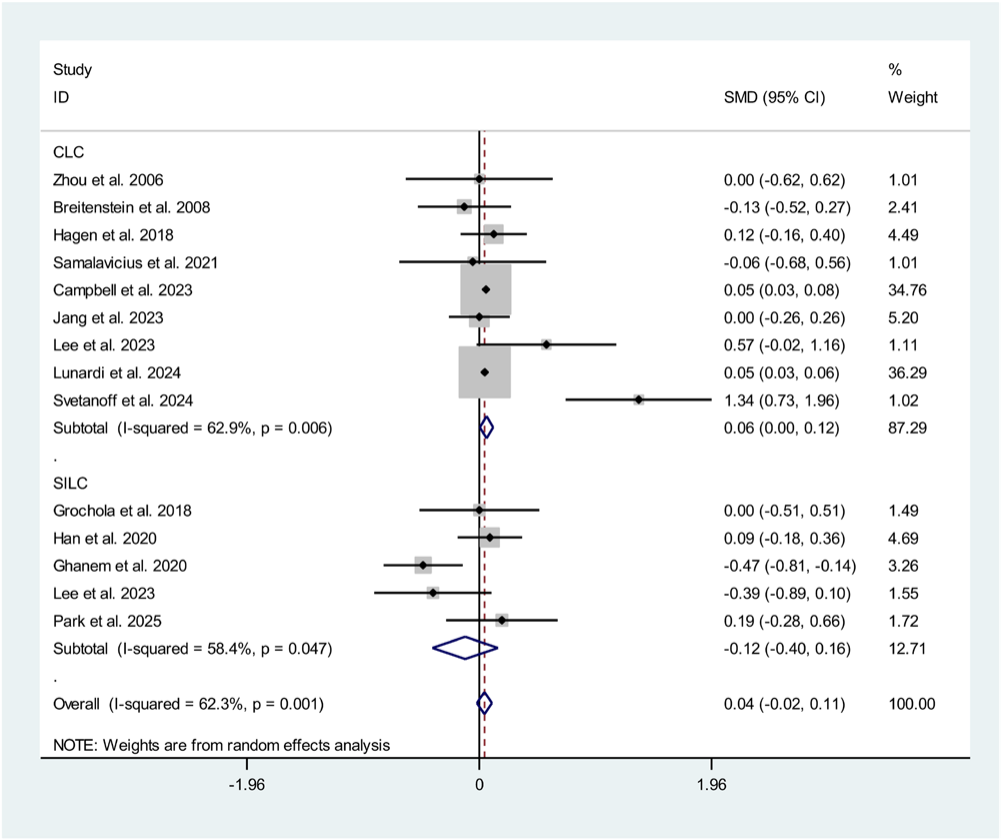
Meta-analysis forest plot concerning the length of hospital stay.

Fourteen studies, including 2 RCTs^[10,13]^ and 12 non-RCTs,^[9,14–22,24,25]^ reported the length of hospital stay. A subgroup analysis for RAC compared with CLC and SILC respectively was performed. Compared with CLC, patients received RAC need a significantly longer length of hospital stay (SMD 0.06 d; 95% CI 0.00, 0.12) with a high heterogeneity (*P* < .01, *I*^2^ = 62.9%). However, compared with SILC, patients received RAC obtained a shorter length of hospital stay (SMD ‐0.12 d; 95% CI ‐0.40, 0.16; *P* = .047, *I*^2^ = 58.4%) though without significant differences. The pooled data showed there was no significant difference for the length of hospital stay between RAC and CLC/SILC (SMD 0.04 d; 95% CI ‐0.02, 0.11) (Fig. 3).

### 3.5. Intraoperative complications

**Figure 4.**
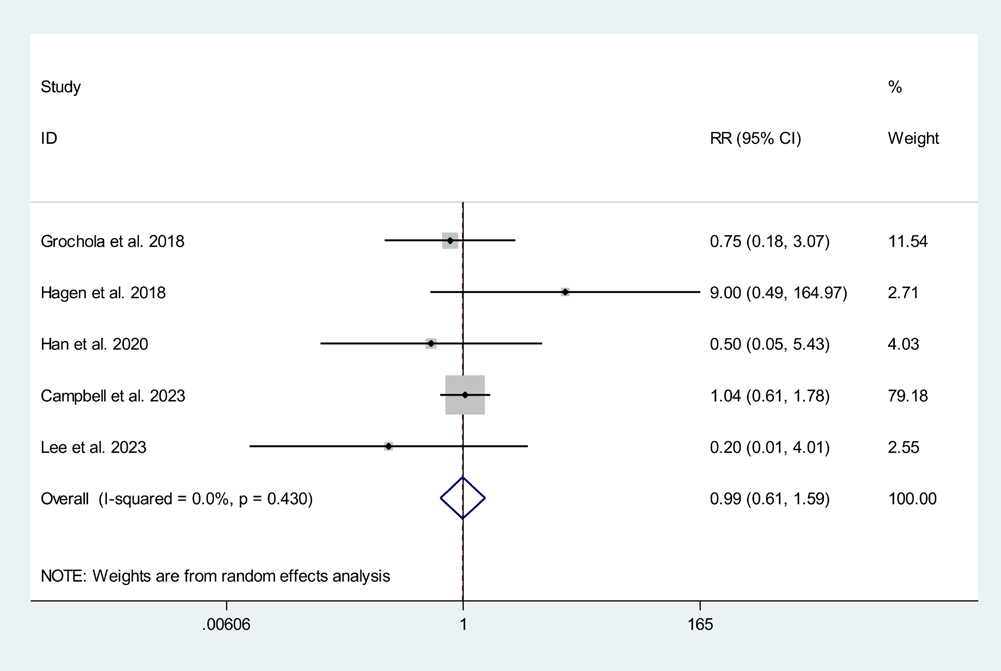
Meta-analysis forest plot concerning intraoperative complications.

Eight studies, including 2 RCTs^[12,13]^ and 6 non-RCTs,^[15,16,20–22,25]^ reported data on intraoperative complications. Of these, 4 studies^[12,21,22,25]^ reported 0 events in the RAC group, and 4 studies^[12,15,21,25]^ reported 0 events in the CLC/SILC group. Meta-analysis showed no significant difference in the rate of intraoperative complication between RAC and CLC/SILC (RR 0.99; 95% CI 0.61, 1.59) without significant statistical heterogeneity (*P* = .430, *I*^2^ = 0.0%) (Fig. 4).

### 3.6. Estimate blood loss

**Figure 5.**
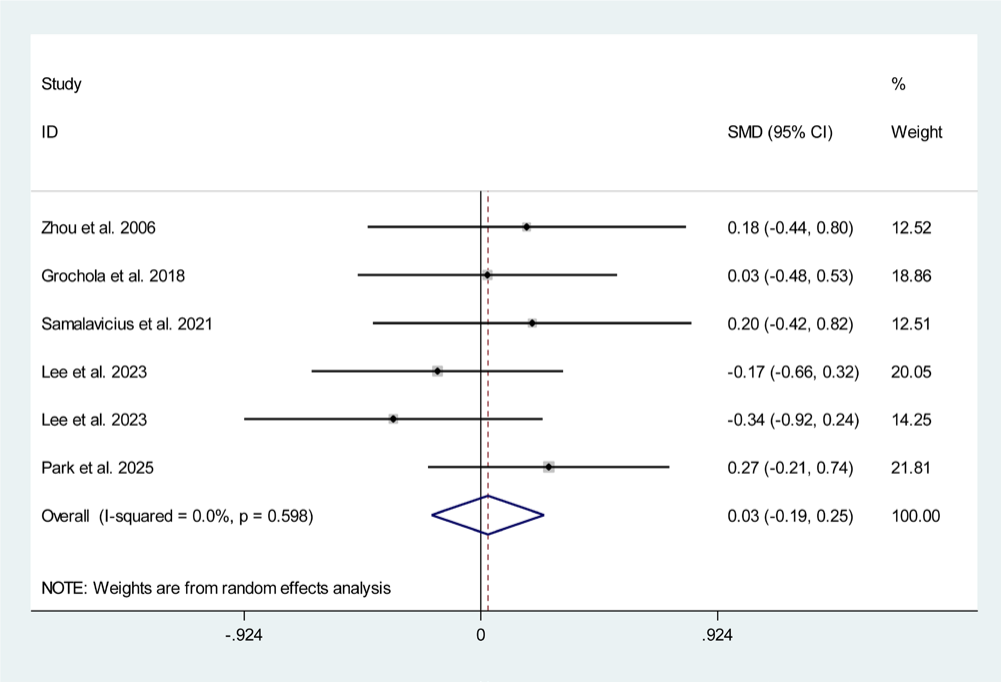
Meta-analysis forest plot concerning estimate blood loss.

Five studies, including 2 RCTs^[10,13]^ and 3 non-RCTs,^[19,22,25]^ reported data on estimate blood loss during operation. Meta-analysis showed no significant difference of estimate blood loss between RAC and CLC/SILC (SMD 0.03 mL; 95% CI ‐0.19, 0.25) without significant statistical heterogeneity (*P* = .598, *I*^2^ = 0.0%) (Fig. 5).

### 3.7. Conversion rate

**Figure 6.**
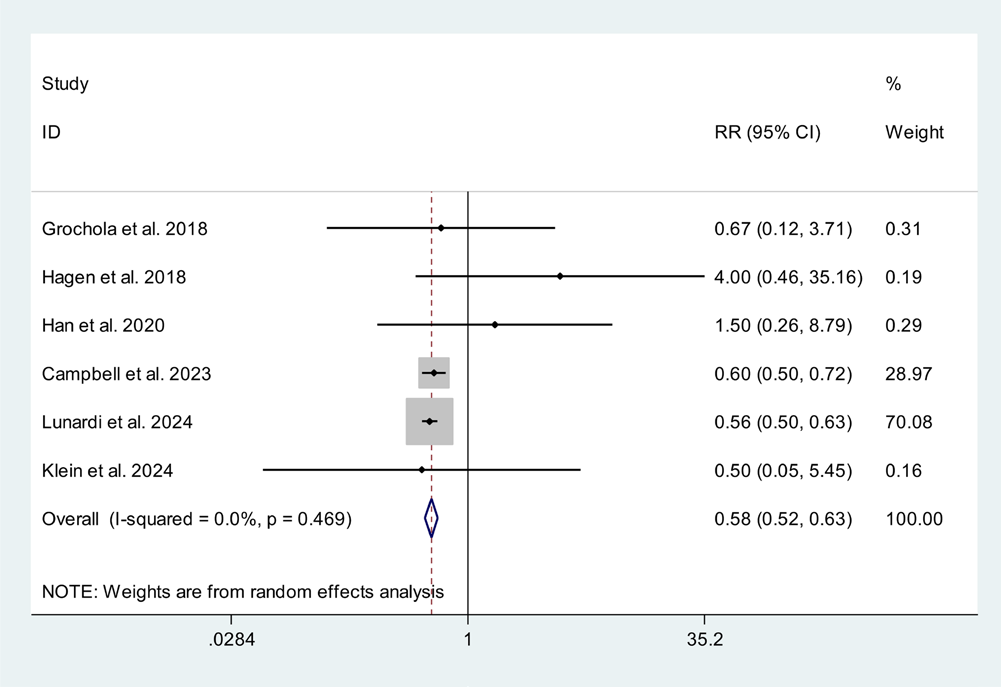
Meta-analysis forest plot concerning conversion rate.

Eleven studies, including 2 RCTs^[11,13]^ and 9 non-RCTs,^[9,14–16,20–23,25]^ reported data on conversion rate. Of these, 5 studies^[11,14,21,22,25]^ reported 0 events in the RAC and CLC/SILC groups. Meta-analysis showed RAC could significantly reduce conversion rate compared with CLC/SILC (RR 0.58; 95% CI 0.52, 0.63) without significant statistical heterogeneity (*P* = .469, *I*^2^ = 0.0%) (Fig. 6).

### 3.8. The rate of incisional hernias

**Figure 7.**
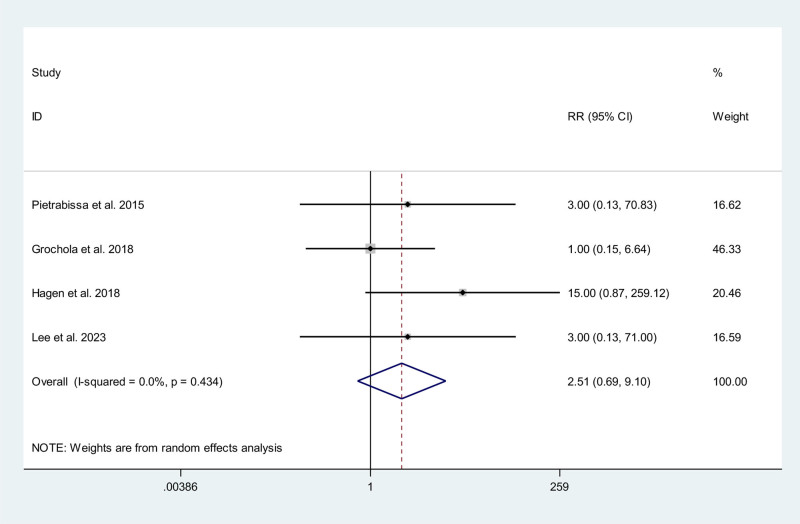
Meta-analysis forest plot concerning the development of incisional hernias.

Four studies, including 2 RCTs^[11,13]^ and 2 non-RCTs,^[15,22]^ reported data on the rate of incisional hernias. Of these, 3 studies^[11,15,22]^ reported 0 events in the CLC/SILC group. Meta-analysis showed the rate of incisional hernias happened in RAC group is higher than that in CLC/SILC group, though the result had no significant difference (RR 2.51; 95% CI 0.69, 9.10) (Fig. 7).

### 3.9. Hospitalization costs

**Figure 8.**
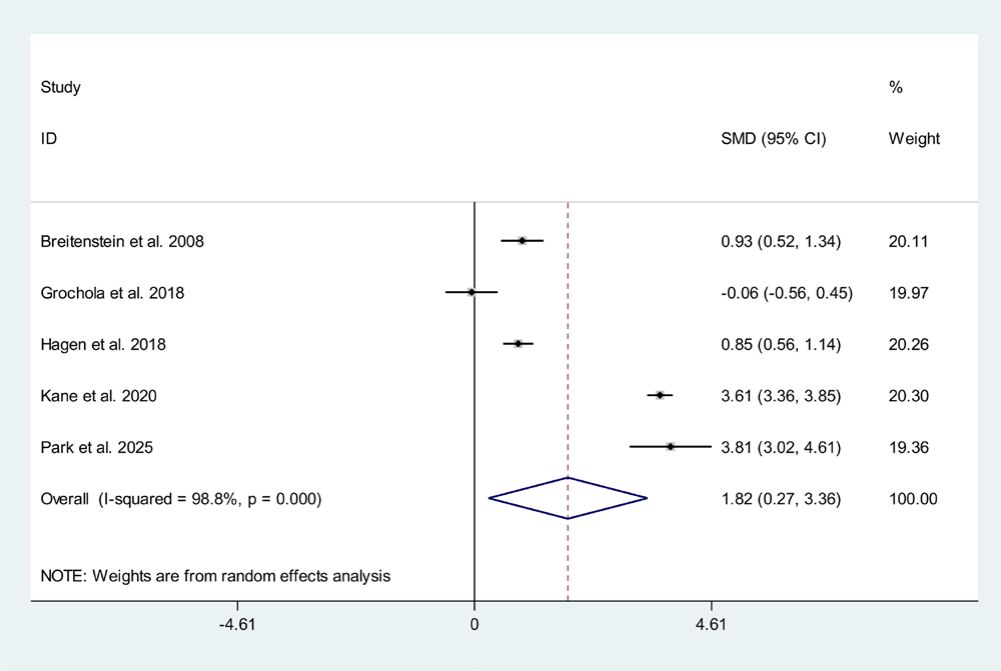
Meta-analysis forest plot concerning the hospitalization costs.

Five studies, including 1 RCTs^[13]^ and 4 non-RCTs,^[14,15,17,25]^ reported data on hospitalization costs. Meta-analysis showed hospitalization costs of RAC is significantly higher than that in CLC/SILC group (SMD 1.82; 95% CI 0.27, 3.36) with a high heterogeneity (*P* < .01, *I*^2^ = 98.8%) (Fig. 8).

### 3.10. Sensitivity analysis

We used leave-one-out sensitivity analysis to evaluate the impact of each study on pooled results and heterogeneity. The outcomes of operative time were stable. There was no effect on statistical significance after removing any individual study. The most significant contribution to heterogeneity from the study by Kane et al^[17]^ (Table S1, Supplemental Digital Content, https://links.lww.com/MD/O965). The outcomes of hospitalization time were stable. The most significant contribution to heterogeneity from the study Svetanoff et al.^[24]^ Omitting Svetanoff et al, the heterogeneity reduced from 62.3% to 31.2% (Table S2, Supplemental Digital Content, https://links.lww.com/MD/O965). The outcomes of hospitalization costs were stable. There was little effect on statistical significance and heterogeneity after removing any individual study (Table S3, Supplemental Digital Content, https://links.lww.com/MD/O965). For other outcomes without heterogeneity, there was no effect on statistical significance after removing any individual study.

### 3.11. Risk of bias assessment

The results of bias assessment for RCTs and non-RCTs involved in the study are presented in Figures S1 and S2, Supplemental Digital Content, https://links.lww.com/MD/O965. For the 4 RCTs involved in the study, 3 showed low risk of bias, 1 showed some concerns because of lack of double-blind procedure during specific randomization process. All non-RCTs showed low overall risk of bias.

The result of publication bias for operative time was shown in Figure S3, Supplemental Digital Content, https://links.lww.com/MD/O965. A symmetrical distribution was obtained in the funnel plot analysis of operative time indicating no publication bias existed. The *P* values of Begg test and Egger test were .06 and .343, respectively.

## 4. Discussion

In this meta-analysis, we included 17 studies comprising 75,866 patients to compare the safety and effectiveness of RAC with those of CLC and SILC in patients with benign gallbladder disease. Our main findings in this study were as follows: (1) RAC was related to longer operative time compared with CLC and SILC. The results had significant difference. (2) RAC could reduce conversion rate to open surgery compared with CLC and SILC. The results had significant difference. (3) RAC was related to higher hospitalization costs compared with CLC and SILC. The results had significant difference. (4) There was no significant difference between groups in the length of hospital stay, rate of intraoperative complications, estimate blood loss and rate of incisional hernias.

Since the first operation for cholecystitis with calculi in 1684, many advancements had been made in surgical management of benign gallbladder disease.^[26]^ In 1987, laparoscopy was first used in medicine, and later in 1991, robots were incorporated.^[27]^ Robotic-assisted laparoscopic cholecystectomy pioneered in 2010 were the newest advancements in biliary surgery.^[28]^ In recent years, RAC had been a practical alternative to traditional laparoscopy and single-incision cholecystectomy for treatment of benign gallbladder disease, with the advantage of reduced postoperative pain, improved cosmetic outcomes and shorter hospital stays. Although it possesses many benefits, RAC comes with certain limitations. Higher hospitalization costs, longer operative time and steeper learning curve associated with robotic technology have raised concerns about its cost-effectiveness. A careful comparison between RAC and CLC/SILC that we performed in this study is necessary.

Different operation time have different effects on the postoperative recovery of patients. Longer operation time is always associated with an increased risk of postoperative complications and cost. In this study, RAC was related to longer operative time compared with CLC/SILC. This finding could be possibly attributed to the learning curve associated with robotic technology and the time required for set up and docking. A meta-analysis published before reported the mean total operative time was 77.29 minutes for RAC. However, RAC procedures such as set up and docking contributed 39.3 ± 12.5 minutes to the mean total operative time. In addition, the operation and docking time decreased as the surgeon gained more experience, cutting the time in half after 2 cases.^[29]^ Meanwhile, the definition of operative time in every study involved in the meta-analysis is not the same. Only 5 studies described the set up and docking time for RAC.^[10,14,19,21,25]^ To reduce heterogeneity of the study, we only included records of skin-to-skin time which was possibly attributed to longer operative time for RAC.

RAC was related to lower rate of conversion rate to open surgery compared with CLC/SILC, which indicates RAC is a safe and effect procedure for benign gallbladder disease. Although laparoscopy can be performed with 1 port, SILC has its own limitations, such as narrow workstations, limited triangulation, and higher complications.^[30]^ The single-site port (22.5-cm vertical incision at the umbilicus) is better suited for robotic-assisted procedures. The Da Vinci robot uses more ergonomic tools with greater ranges of motion, thereby allowing triangulation to be restored by separating the tools beyond the port entry and at the working end.^[31]^ Other advantages of Da Vinci robot such as 3D vision, magnification and clearer visibility also make cholecystectomy safer for the patients on average.^[28]^

A significant issue in operative cost remains unresolved. Laparoscopic equipment has a relatively affordable cost, while the Da Vinci robot costs more than 1,500,000 Euros, plus 150,000 Euros per yearly service. Robotic-assisted surgery remains high-priced compared with laparoscopic surgery, which was also confirmed in this study. As robotic-assisted surgery become more and more popular, new robotic systems are also invented in recent years. Sooner, the operative cost will be more reasonable.^[32,33]^ Patient safety, outcomes, and surgeon’s convenience are mandatory.

There was no significant difference between groups in the length of hospital stay, rate of intraoperative complications, estimate blood loss and rate of incisional hernias. Compared with conventional 4-port or 3-port laparoscopic cholecystectomy, RAC has fewer incision wound, which reduced postoperative pain and improved cosmetic outcomes. Compared with SILC, RAC was much more convenient for cholecystectomy while with the same incision wound. As more and more surgeons use robotic system to perform cholecystectomy, a shorter length of hospital stay, and lower rate of intraoperative complications are available in future.

This study has some limitations as follows: (1) There are only 4 RCTs meeting the inclusion criteria. To obtain a reliable conclusion, we included 13 high-quality observational studies with PSM. More RCTs comparing RAC and CLC/SILC are needed in future. (2) The definition of operative time for RAC is still controversial. As more and more surgeons accept robotic-assisted surgery, the operative time of RAC should be redefined and recorded. (3) Since most RAC was performed in single-site port, comparison between RAC and SILC is more meaningful. However, there were still lack of high-quality studies about RAC and SILC.

## 5. Conclusion

RAC was related to longer operative time and reduced conversion rate to open surgery compared with CLC and SILC. There was no significant difference between groups in the length of hospital stay, rate of intraoperative complications, estimate blood loss and rate of incisional hernias. In conclusion, RAC is a safe and effective procedure with lower conversion rate and comparable rate of complications compared with CLC/SILC. A significant issue in operative cost remains unresolved.

## Author contributions

**Conceptualization:** Kezhong Tang, Yizhao Zhou.

**Data curation:** Kezhong Tang.

**Formal analysis:** Kezhong Tang.

**Funding acquisition:** Wei Zhou.

**Investigation:** Wei Zhou, Yongzhou Li.

**Methodology:** Wei Zhou.

**Resources:** Yizhao Zhou.

**Software:** Yizhao Zhou.

**Validation:** Yongzhou Li, Li Liao.

**Visualization:** Yongzhou Li, Li Liao, Xin Dong.

**Supervision:** Li Liao.

**Writing—original draft:** Xin Dong.

**Writing—review & editing:** Xin Dong.

## Supplementary Material


