# Supplementary material for: Robotic-assisted versus conventional/single-incision laparoscopic cholecystectomy for benign gallbladder disease: A systematic review and meta-analysis
Source: Medicine (Baltimore). 2025 May 23;104(21):e42493. doi: 10.1097/MD.0000000000042493 (PMC12114010; doi:10.1097/MD.0000000000042493)
Supplement: Supplementary file 1 [file medi-104-e42493-s001.pdf]

# Supplementary material

Table S1 Leave-one-out sensitivity analysis for the operative time outcome.

| study | SDM | 95% CI | I2 |
|---|---|---|---|
| Omitting Kane et al. 2020 | 0.57 | 0.33, 0.81 | 90.60% |
| Omitting Zhou et al. 2006 | 0.81 | 0.30, 1.32 | 98.30% |
| Omitting Breitenstein et al. 2008 | 0.83 | 0.31, 1.35 | 98.30% |
| Omitting Pietrabissa et al. 2015 | 0.82 | 0.30, 1.33 | 98.30% |
| Omitting Kudsi et al. 2017 | 0.8 | 0.27, 1.32 | 98.30% |
| Omitting Grochola et al. 2018 | 0.81 | 0.30, 1.33 | 98.30% |
| Omitting Hagen et al. 2018 | 0.83 | 0.30, 1.37 | 98.30% |
| Omitting Han et al. 2020 | 0.71 | 0.21, 1.20 | 98.10% |
| Omitting Ghanem et al. 2020 | 0.84 | 0.32, 1.37 | 98.30% |
| Omitting Samalavicius et al. 2021 | 0.76 | 0.25, 1.27 | 98.30% |
| Omitting Campbell et al. 2023 | 0.82 | 0.16, 1.48 | 98.00% |
| Omitting Jang et al. 2023 | 0.79 | 0.26, 1.33 | 98.30% |
| Omitting Lee et al. 2023 | 0.75 | 0.22, 1.28 | 98.40% |
| Omitting Klein et al. 2024 | 0.85 | 0.30, 1.39 | 98.30% |
| Omitting Svetanoff et al. 2024 | 0.83 | 0.32, 1.35 | 98.30% |
| Omitting Park et al. 2025 | 0.8 | 0.29, 1.32 | 98.30% |

Table S2 Leave-one-out sensitivity analysis for the hospitalization time outcome.

| study | SDM | 95% CI | I2 |
|---|---|---|---|
| Omitting Zhou et al. 2006 | 0.04 | -0.02, 0.11 | 65.20% |
| Omitting Breitenstein et al. | 0.05 | -0.02, 0.11 | 64.40% |

| | | | |
|---|---|---|---|
| 2008 | | | |
| Omitting Grochola et al. 2018 | 0.04 | -0.02, 0.11 | 65.20% |
| Omitting Hagen et al. 2018 | 0.04 | -0.03, 0.10 | 64.90% |
| Omitting Han et al. 2020 | 0.04 | -0.02, 0.11 | 65.10% |
| Omitting Ghanem et al. 2020 | 0.06 | 0.00, 0.11 | 52.10% |
| Omitting Samalavicius et al. 2021 | 0.04 | -0.02, 0.11 | 65.10% |
| Omitting Campbell et al. 2023 | 0.05 | -0.10, 0.20 | 64.90% |
| Omitting Jang et al. 2023 | 0.05 | -0.02, 0.11 | 65.10% |
| Omitting Lee et al. 2023 | 0.04 | -0.01, 0.10 | 61.40% |
| Omitting Lunardi et al. 2024 | 0.05 | -0.10, 0.20 | 65.00% |
| Omitting Svetanoff et al. 2024 | 0.04 | 0.00, 0.08 | 31.20% |
| Omitting Park et al. 2025 | 0.04 | -0.02, 0.10 | 64.80% |

Table S3 Leave-one-out sensitivity analysis for the hospitalization costs outcome.

| study | SDM | 95% CI | I2 |
|---|---|---|---|
| Omitting Breitenstein et al. 2008 | 2.04 | 0.15, 3.94 | 99.00% |
| Omitting Grochola et al. 2018 | 2.29 | 0.61, 3.96 | 98.80% |
| Omitting Hagen et al. 2018 | 2.07 | 0.13, 4.00 | 98.80% |
| Omitting Kane et al. 2020 | 1.33 | 0.27,2.39 | 95.40% |
| Omitting Park et al. 2025 | 1.34 | -0.39, 3.07 | 99% |

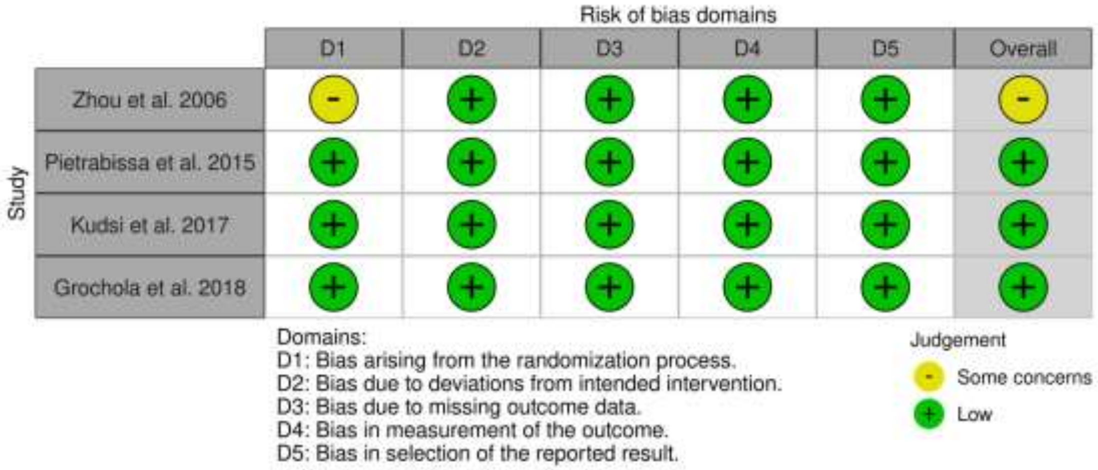


Fig. S1 Assessment according to the Cochrane Collaboration’s tool for evaluating risk of bias in randomized trials (Rob-2)

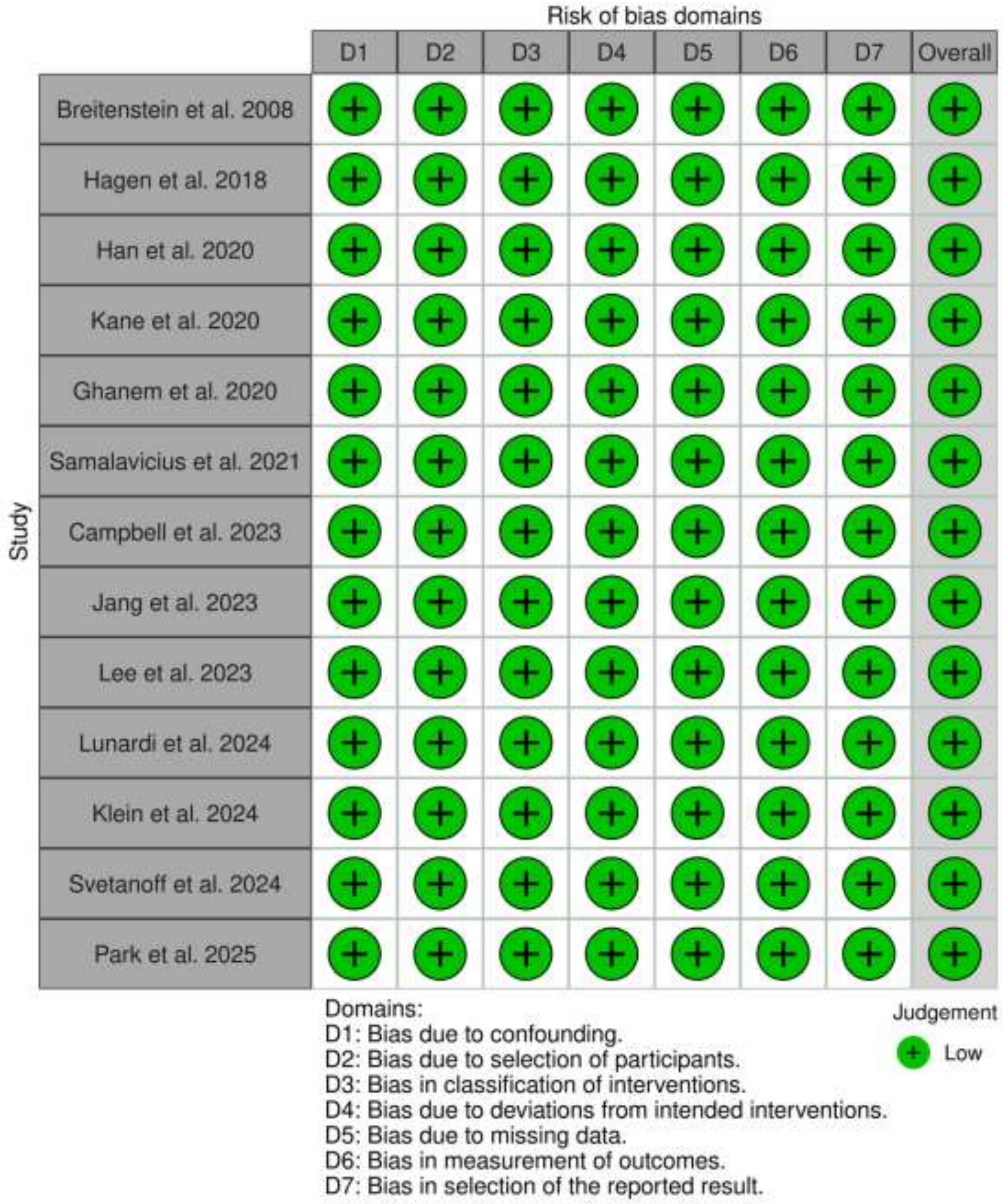


Fig. S2 Assessment according to the Cochrane Collaboration's tool for evaluating risk of bias in non-randomized trials (ROBINS-I)

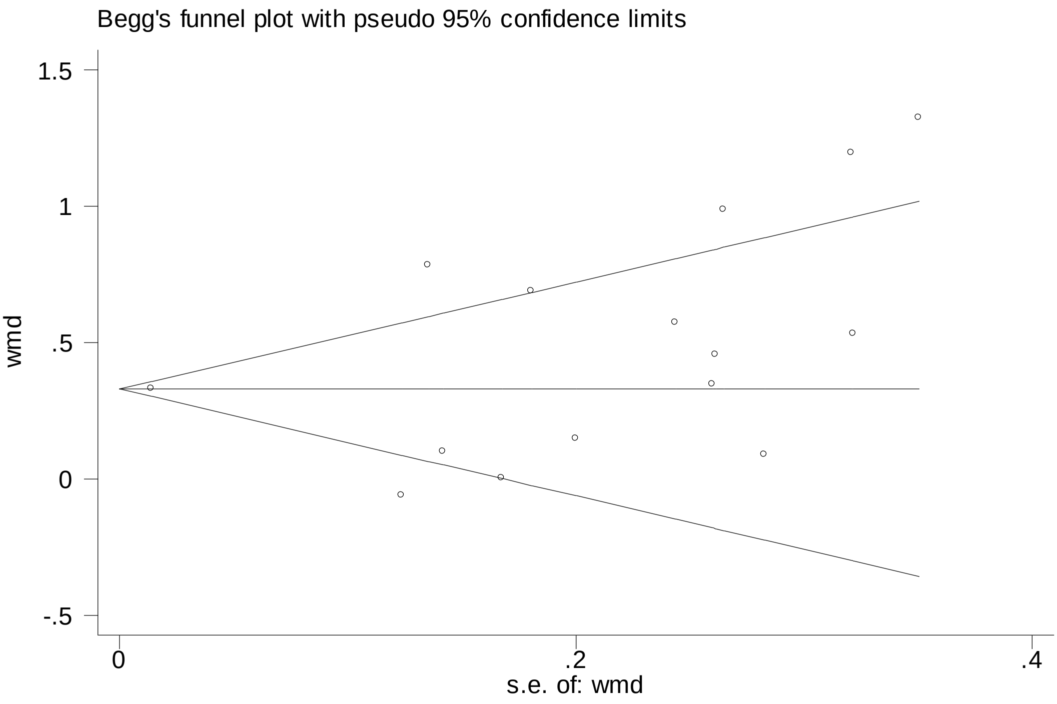


Fig. S3 Funnel plot of operative time
